# Author Correction: New carbohydrate binding domains identified by phage display based functional metagenomic screens of human gut microbiota

**DOI:** 10.1038/s42003-024-06920-0

**Published:** 2024-09-26

**Authors:** Akil Akhtar, Madhu Lata, Sonali Sunsunwal, Amit Yadav, Kajal LNU, Srikrishna Subramanian, T. N. C. Ramya

**Affiliations:** 1 CSIR- Institute of Microbial Technology, Sector 39-A, Chandigarh, 160036 India; 2 Academy of Scientific & Innovative Research (AcSIR), Ghaziabad, Uttar Pradesh 201002 India; 3 Present Address: Emory Vaccine Center, Emory University, Atlanta, GA USA

**Keywords:** Glycobiology, Dietary carbohydrates, Lectins, High-throughput screening, Metagenomics

Correction to: *Communications Biology* 10.1038/s42003-023-04718-0, published online 5 April 2023

The original version of the article contained an error in the text which should be “translation frame 3” instead of “translation frame 2” in the below mentioned sentence in the Results section:

“However, the amino acid sequence from translation frame 3 of the ORF (listed as MF12F2 in Supplementary Data 1, Table S4) returned transposase proteins as top hits (Supplementary Data 1, Table S5).”

This has now been corrected in the PDF and HTML versions of the Article.

