# Supplementary material for: New carbohydrate binding domains identified by phage display based functional metagenomic screens of human gut microbiota
Source: Commun Biol. 2023 Apr 5;6:371. doi: 10.1038/s42003-023-04718-0 (PMC10076258; doi:10.1038/s42003-023-04718-0)
Supplement: Supplementary file 2 — Supplementary Figures S1 to S11 [file 42003_2023_4718_MOESM2_ESM.pdf]

SUPPLEMENTARY FIGURES S1-S11

New carbohydrate binding domains identified by phage display based functional metagenomic screens of human gut microbiota

Akil Akhtar[1,3], Madhu Lata[1,2], Sonali Sunsunwal[1,4], Amit Yadav[1,2,4], Kajal[1,2,4], Srikrishna Subramanian[1,2] and T.N.C. Ramya[1,2,*]

[1]CSIR- Institute of Microbial Technology, Sector 39-A, Chandigarh 160036, INDIA

[2]Academy of Scientific & Innovative Research (AcSIR), Ghaziabad, Uttar Pradesh 201002, INDIA

[3]Currently at Emory Vaccine Center, Emory University, Atlanta, GA, United States of America

[4]These authors contributed equally

[*]Correspondence to be addressed to

T.N.C. Ramya, CSIR- Institute of Microbial Technology, Sector 39-A, Chandigarh 160036, INDIA. Tel: 91-172-2880243; E-mail: ramya@imtech.res.in

Running Title: Carbohydrate binding domains from a metagenomic human fecal phage display library

Figure S1

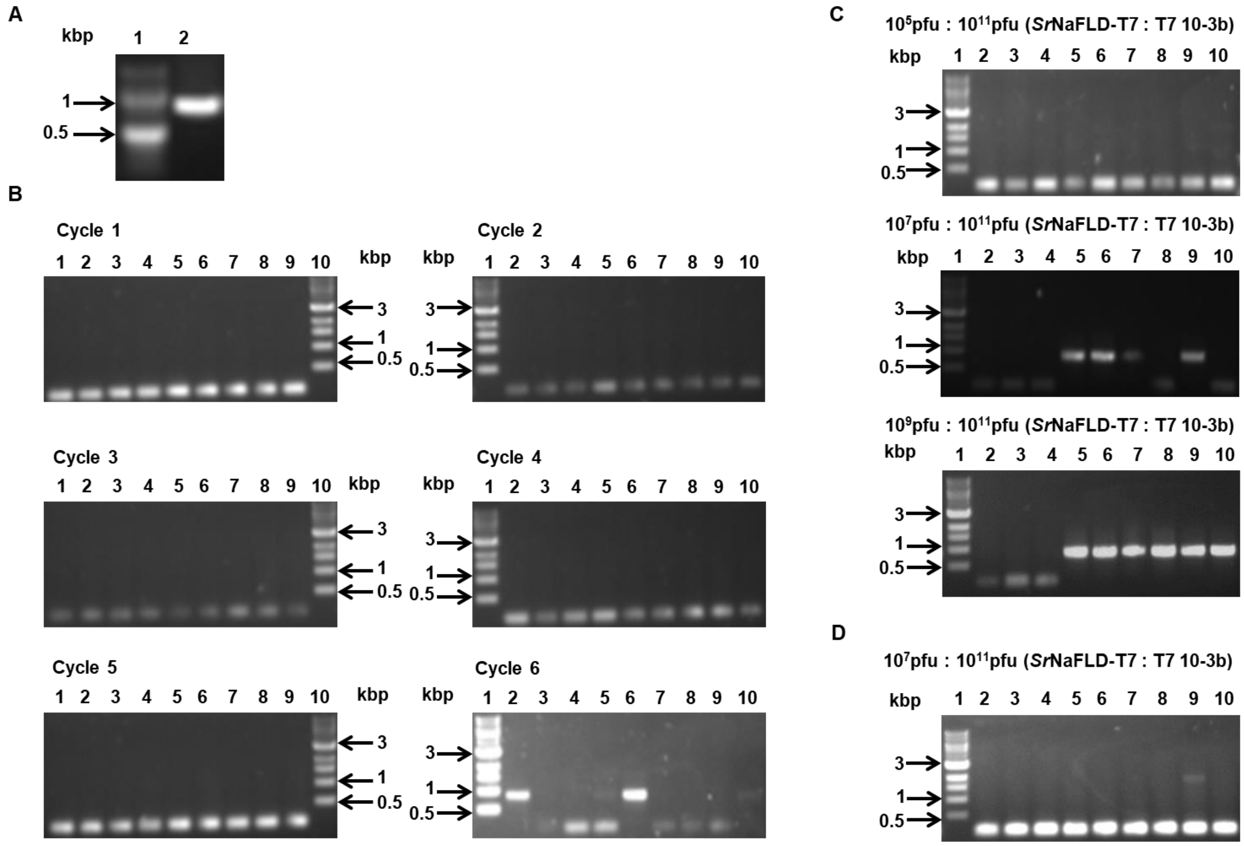

Generation of *Sr*NaFLD recombinant phages and optimization of screening protocol. (A) Agarose gel electrophoresis indicating the amplicon in *Sr*NaFLD recombinant T7 phage. Lane 1: 1 kbp DNA marker (NEB). Lane 2: PCR amplicon amplified from the *Sr*NaFLD T7 phage DNA. (B) Agarose gel electrophoresis of PCR amplicons indicating the presence or absence of the *Sr*NaFLD DNA insert in phages extracted from nine plaques randomly selected following each of the six cycles of the biopanning procedure performed. The biopanning was initiated with 100 µl of a phage suspension with a titer of ~10^{11} pfu/ml containing *Sr*NaFLD recombinant phages and non-recombinant T7 phages in a ratio $1x10^{3}$: $1x10^{11}$ (*Sr*NaFLD-T7 phage: T7Select 10-3b phage). Lane 10 in gels of cycles 1, 3 and 5, and lane 1 in gels of cycles 2, 4, and 6 have 1 kbp DNA molecular marker. DNA bands corresponding to *Sr*NaFLD DNA insert (~900 bp) are observed in phage plaques following biopanning in cycle 6. (C) Agarose gel electrophoresis indicating the results of the fourth round of the biopanning of 100 µl of a phage suspension with a titer of ~10^{11} pfu/ml containing *Sr*NaFLD recombinant phages and non-recombinant T7 phages in different ratios $1x10^{5}$: $1x10^{11}$, $1x10^{7}$: $1x10^{11}$, $1x10^{9}$: $1x10^{11}$ (*Sr*NaFLD-T7 phage: T7 10-3b phage). Lane 1: 1 kbp DNA marker. Lanes 2-10: PCR amplicons of the phages extracted from 9 different plaques after biopanning. (D) Agarose gel electrophoresis indicating the results of the biopanning of 100 µl of a phage suspension with a titer of ~10^{11} pfu/ml containing *Sr*NaFLD recombinant phages and non-recombinant T7 phages in ratio $1x10^{7}$: $1x10^{11}$ (*Sr*NaFLD-T7 phage: T7 10-3b phage) with 1%SDS used as the elution agent. Lane 1: 1 kbp DNA marker. Lanes 2-10: PCR products of the phages extracted from 9 different plaques after 6 rounds of biopanning.

Figure S2

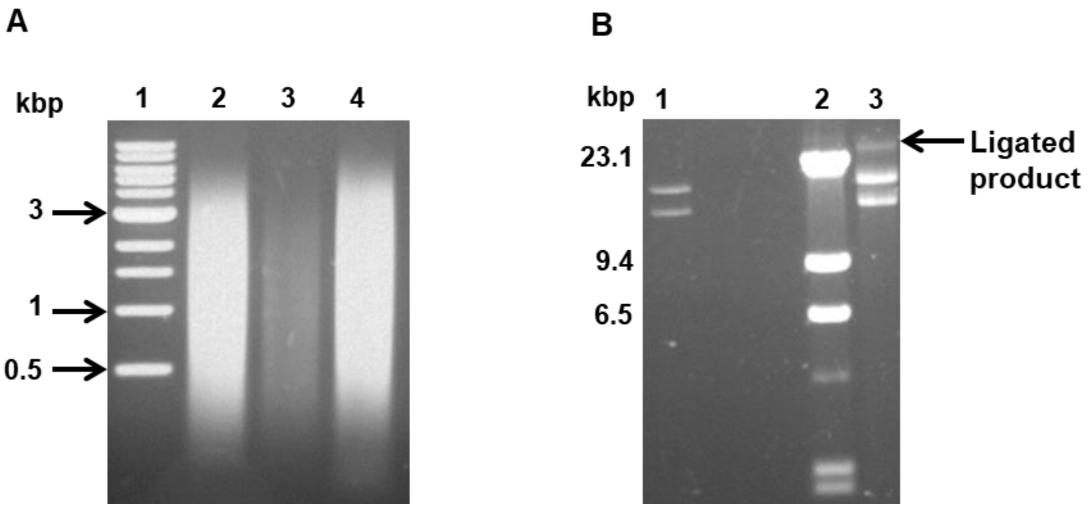

Construction of metagenomic phage display library. (A) Agarose gel electrophoresis indicating fragmentation of metagenomic DNA. Lane 1: 1 kbp DNA marker from NEB. Lanes 2-4: Fragments of 500 to 300 base pairs of the metagenomic DNA isolated from different kits produced by sonication. (B) Agarose gel electrophoresis indicating ligation of metagenomic fragments into T7Select 10-3b vector. Lane 1: SmaI (NEB) digested T7Select 10-3b vector DNA. Lane 2: λ DNA-HindIII Digest (marker DNA) from NEB. Lane 3: Ligation reaction mix containing a band >23.1 kbp confirming the blunt end ligation of the fragmented metagenomic DNA into the T7Select 10-3b vector.

Figure S3

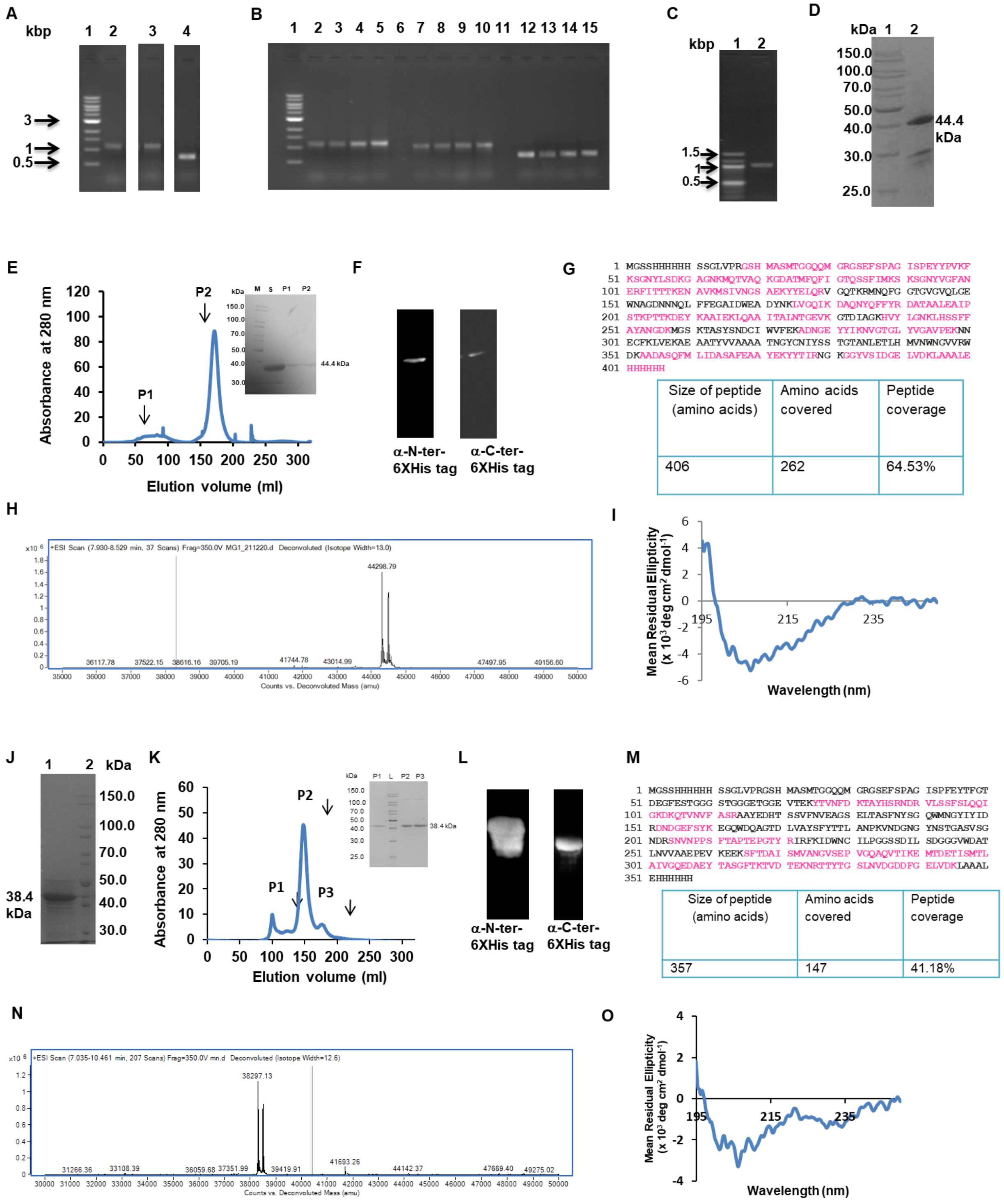

G:

Size of peptide (amino acids)	Amino acids covered	Peptide coverage
406	262	64.53%

M:

Size of peptide (amino acids)	Amino acids covered	Peptide coverage
357	147	41.18%

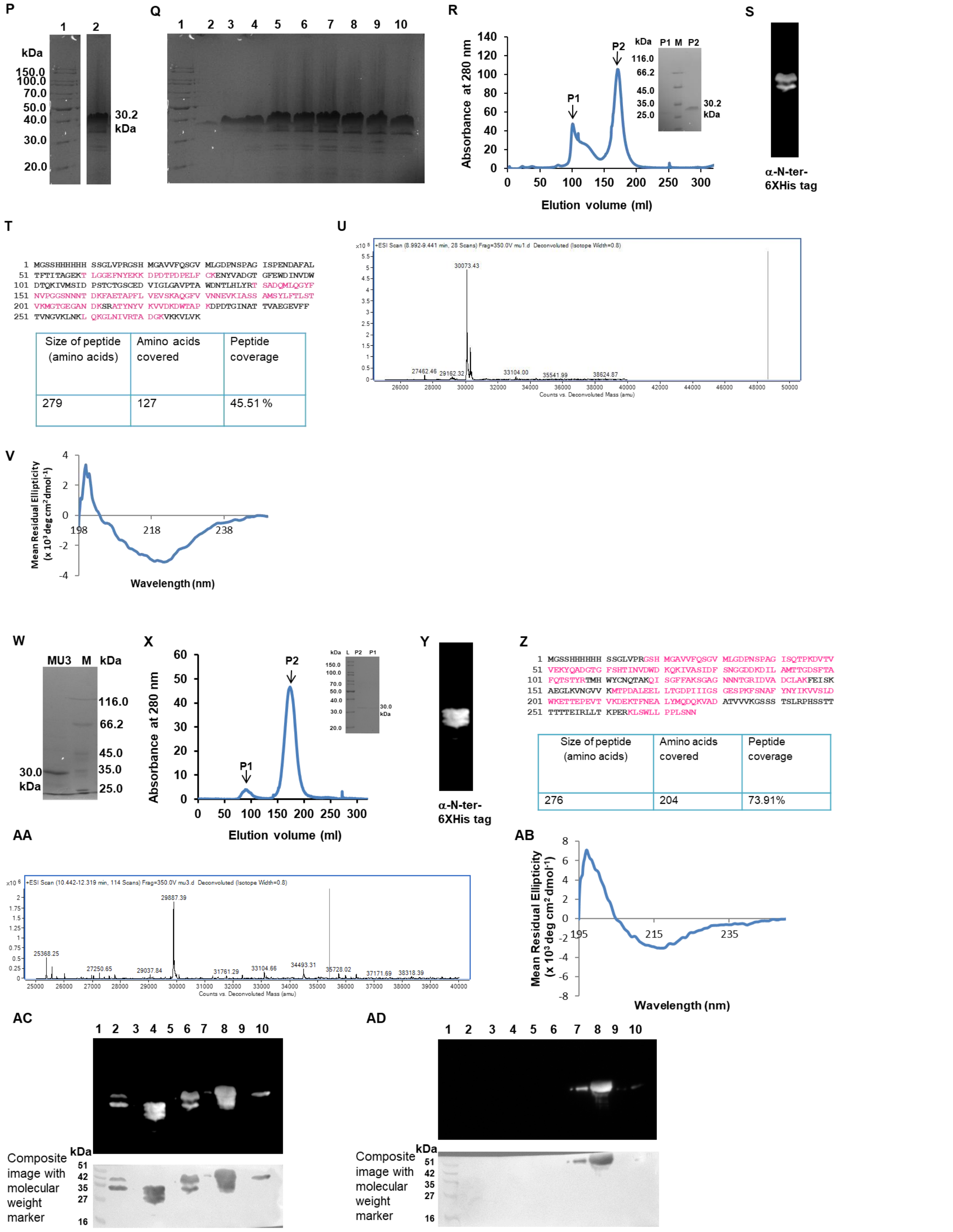

Cloning, expression, and purification of MG1 (A-G), MN3 (H-N), MU1 (O-U), and MU3 (V-AB). (A) PCR amplification. Lane 1: DNA marker, Lane 2: PCR amplicon of MG1 clone. Lane 3:

PCR amplicon of MU1 clone. Lane 4: PCR amplicon of MU3 clone. (B) Uncropped image pertaining to Figure S3A. Lanes 9 and 12 have been sliced out to form lanes 3 and 4 of S3A. (C) PCR amplification. Lane 1: DNA marker, Lane 2: PCR amplicon of MN3 clone. (D) Coomassie stained SDS-PAGE. Lane 1: Molecular mass marker, Lane 2: Purified recombinant MG1 protein. (E) Size Exclusion Chromatogram of MG1 protein using Hiprep 26/60 Sephacryl S-200 High Resolution column with eluted peak fractions P1 and P2, and input protein sample S analyzed by SDS-PAGE in inset. (F) Western analysis of recombinant MG1 protein using N-terminal anti-His antibody and C-terminal anti-His antibody. (G) Sequence coverage obtained with MS/MS spectrometry of recombinant MG1 protein followed by targeted sequence search; the peptide sequence coverage is shown in pink color. Results of a single mass spectrometry run. (H) Deconvoluted spectrum showing the intact masses of recombinant MG1 protein using Agilent G6550A Quadrupole Time of Flight (Q-TOF) mass spectrometer. MG1 theoretical molecular mass: 44.43 kDa; observed molecular masses of 44.3 kDa and 44.478 kDa correspond to protein with N-terminal methionine cleavage and cysteine oxidation. (I) Circular dichroism spectrum of recombinant MG1 protein. (J) Coomassie stained SDS-PAGE. Lane 1: Purified recombinant MN3 protein, Lane 2: Molecular mass marker. (K) Size Exclusion Chromatogram of MN3 protein using Hiprep 26/60 Sephacryl S-200 High Resolution column with eluted peak fractions P1, P2, and P3 analyzed by SDS-PAGE in inset. (L) Western analysis of recombinant MN3 protein using N-terminal anti-His antibody and C-terminal anti-His antibody. (M) Sequence coverage obtained with MS/MS spectrometry of recombinant MN3 protein followed by targeted sequence search; the peptide sequence coverage is shown in pink color. Results of a single mass spectrometry run. (N) Deconvoluted spectrum showing the intact masses of recombinant MN3 protein using Agilent G6550A Quadrupole Time of Flight (Q-TOF) mass spectrometer. MN3 theoretical molecular mass: 38.427 kDa; observed molecular masses of 38.297 kDa and 38.475 kDa correspond to protein with N-terminal methionine cleavage and cysteine oxidation. (O) Circular dichroism spectrum of recombinant MN3 protein. (P) Coomassie stained SDS-PAGE. Lane 1: Molecular mass marker, Lane 2: Purified recombinant MU1 protein. (Q) Uncropped image pertaining to Figure S3P. Lanes 1 and 6 have been sliced out to form lanes 1 and 2 of S3P. (R) Size Exclusion Chromatogram of MU1 protein using Hiprep 26/60 Sephacryl S-200 High Resolution column with eluted peak fractions P1 and P2 analyzed by SDS-PAGE in inset. (S) Western analysis of recombinant MU1 protein using N-terminal anti-His antibody. (T) Sequence coverage obtained with MS/MS spectrometry of recombinant MU1 protein followed by targeted sequence search; the peptide sequence coverage is shown in pink color. Results of a single mass spectrometry run. (U) Deconvoluted spectrum showing the intact masses of recombinant MU1 protein using Agilent G6550A Quadrupole Time of Flight (Q-TOF) mass spectrometer. MU1 theoretical molecular mass: 30.203 kDa; observed molecular mass of 30.073 kDa corresponds to protein with N-terminal methionine cleavage. (V) Circular dichroism spectrum of recombinant MU1 protein. (W) Coomassie stained SDS-PAGE. Lane 1: Molecular mass marker, Lane 2: Purified recombinant MU3 protein. (X) Size Exclusion Chromatogram of MU3 protein using Hiprep 26/60 Sephacryl S-200 High Resolution column with eluted peak fractions P1 and P2 analyzed by SDS-PAGE in inset. (Y) Western analysis of recombinant MU3 protein using N-terminal anti-His antibody. (Z) Sequence coverage obtained with MS/MS spectrometry of recombinant MU3 protein followed by targeted sequence search; the peptide sequence coverage is shown in pink color. Results of a single mass spectrometry run. (AA) Deconvoluted spectrum showing the intact masses of recombinant MU3 protein using Agilent G6550A Quadrupole Time of Flight (Q-TOF) mass spectrometer. MU3 theoretical molecular mass: 30.0017 kDa, observed molecular mass of 29.887 kDa corresponds to protein with N-terminal methionine cleavage. (AB) Circular dichroism spectrum of recombinant MU3 protein. (AC) Uncropped images pertaining to anti-N-ter-6XHis tag antibody stained blot in Figures S3F, S3L, S3S, and S3Y (lanes 10, 8, 6, and 4, respectively). (AD) Uncropped images pertaining to anti-C-ter-6XHis tag antibody stained blot in Figures S3F and S3L (lanes 10 and 8, respectively).

Figure S4

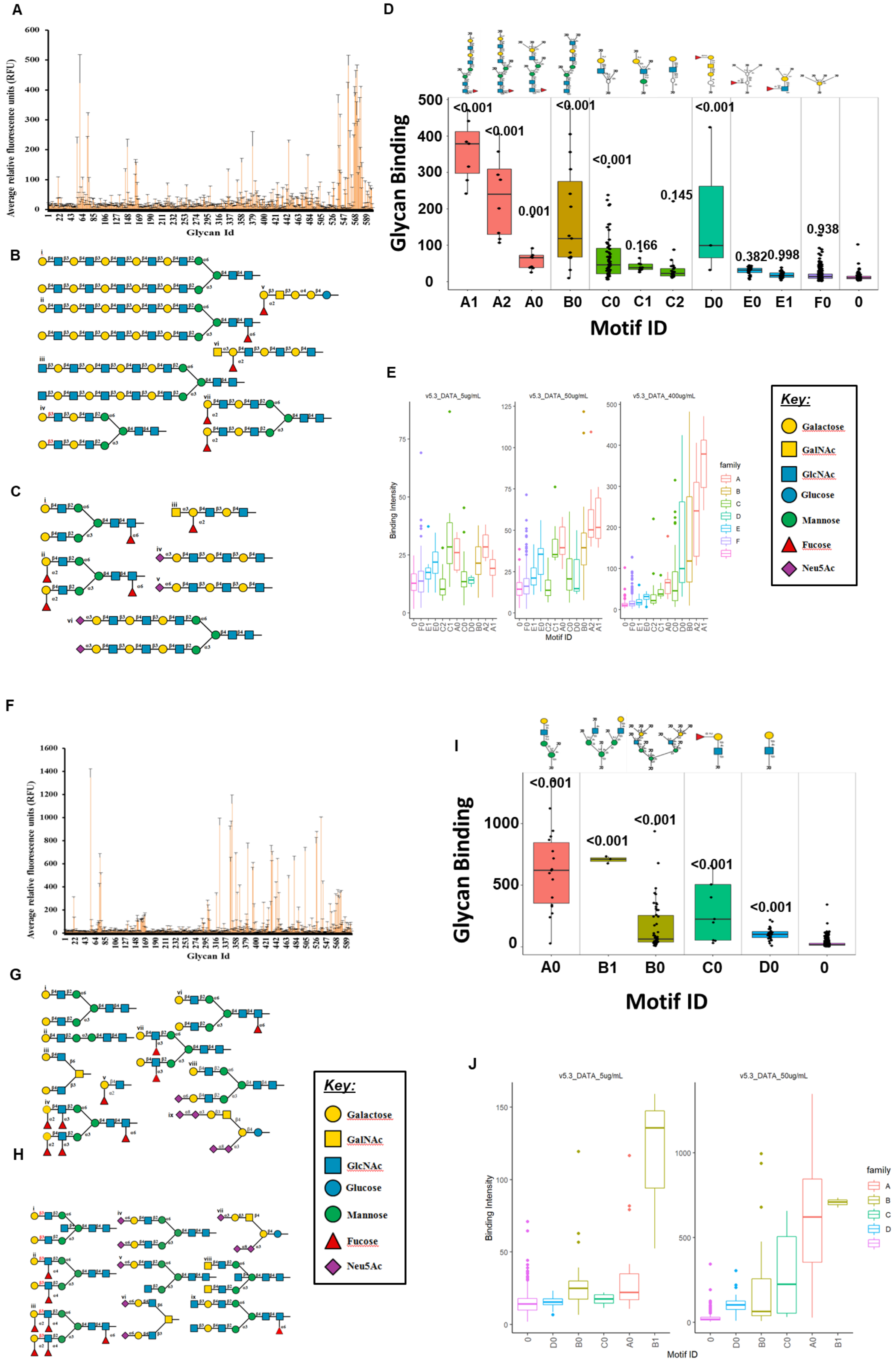

Glycan Binding
Motif ID
Glycan Id
Average relative fluorescence units (RFU)
Key:
Galactose
GalNAc
GlcNAc
Glucose
Mannose
Fucose
Neu5Ac
v5.3_DATA_5ug/mL
v5.3_DATA_50ug/mL
v5.3_DATA_400ug/mL
Binding Intensity
family

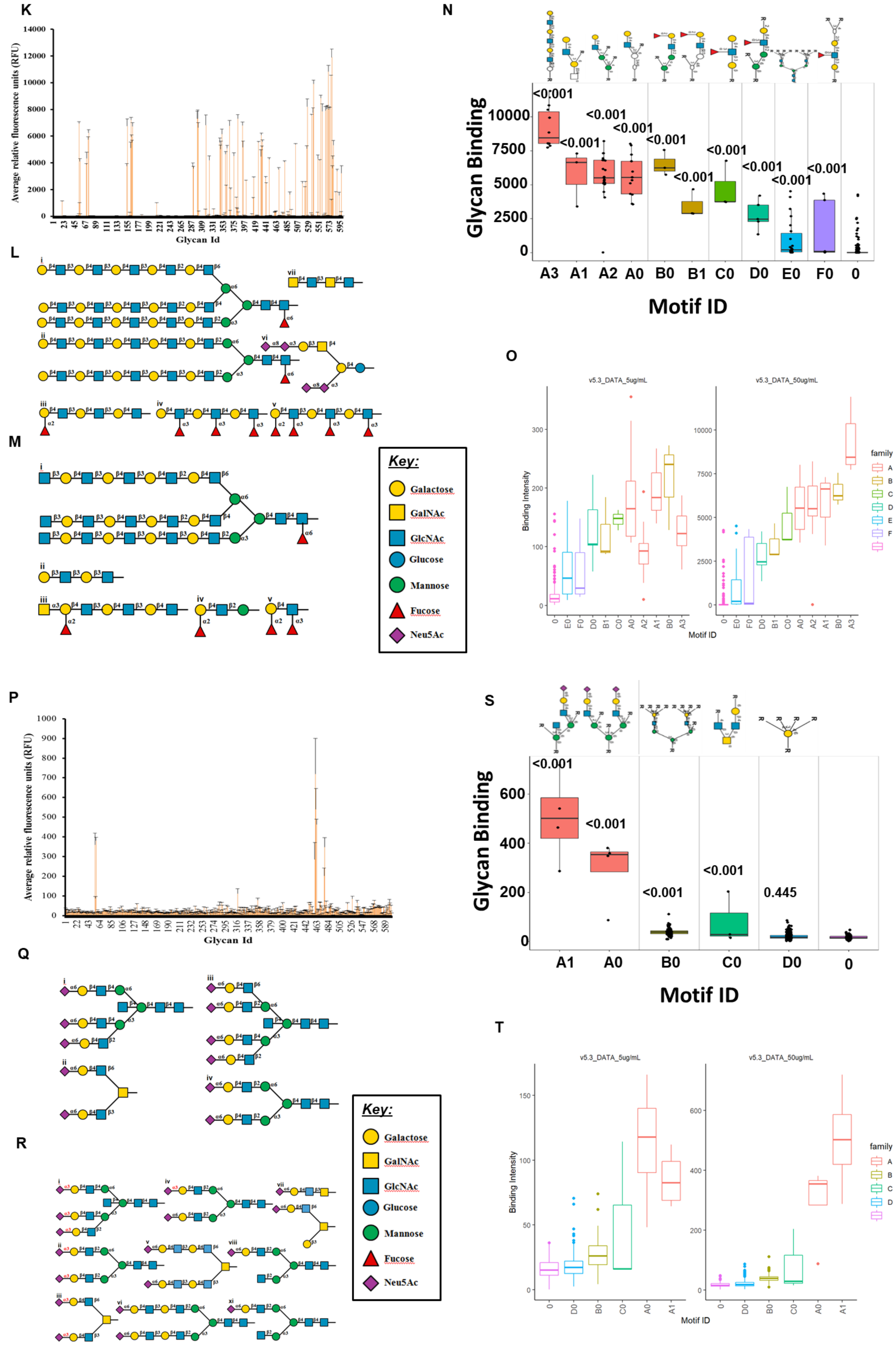
K
Average relative fluorescence units (RFU)
Glycan Id
L
M
Key:
Galactose
GalNAc
GlcNAc
Glucose
Mannose
Fucose
Neu5Ac
N
Glycan Binding
Motif ID
A3 A1 A2 A0 B0 B1 C0 D0 E0 F0 0
<0.001
O
v5.3_DATA_5ug/mL
v5.3_DATA_50ug/mL
Binding Intensity
Motif ID
family
P
Average relative fluorescence units (RFU)
Glycan Id
Q
R
Key:
Galactose
GalNAc
GlcNAc
Glucose
Mannose
Fucose
Neu5Ac
S
Glycan Binding
Motif ID
A1 A0 B0 C0 D0 0
<0.001
0.445
T
v5.3_DATA_5ug/mL
v5.3_DATA_50ug/mL
Binding Intensity
Motif ID
family

Glycan binding by purified recombinant MG1 (A-E), MN3 (F-J), MU1 (K-O), and MU3 (P-T). (A) Bar plot showing the glycan binding profile of MG1. Glycan array analysis was performed on CFG microarray v5.3 with 400 µg/ml MG1. Results of a single glycan array experiment. Spots with highest and lowest fluorescence intensity (of the eight spots for each glycan) were removed. Error bars represent standard deviations. (B) Glycans found to be bound by MG1 (representative glycans out of the 39 glycans found to be bound by MG1). Results of Glycopattern tool using three glycan array datasets of the same protein preparation at 5, 50 and 400 µg/ml. (i-ii) Glycans with terminal Galactose (the top two binders); (iii) Glycan with terminal GlcNAc; (iv) Glycan having type-1 extension unit; (v) Glycan with type-4 H antigen; (vi) Glycan with type-2 A antigen; (vii) Glycan with type-2 H antigen. (C) Glycans found not to bind to MG1. Results of Glycopattern tool using three glycan array datasets of the same protein preparation at 5, 50 and 400 µg/ml. (i-iii) Glycans with short LacNAc chains. (iv-vi) Glycans having terminal acidic residues. Monosaccharide symbols follow the SNFG (Symbol Nomenclature for Glycans) system (Varki, A., et al. 2015). (D) Glycan motifs identified by Motif Finder using automated model building function and default motif list and model options. Motif Finder analysis was performed using three glycan array datasets of the same protein preparation at 5, 50 and 400 µg/ml. (E) Concentration dependent binding of MG1 to glycan motifs identified by Motif Finder. Results of Motif Finder analysis using three glycan array datasets of the same protein preparation at 5, 50 and 400 µg/ml. (F) Bar plot showing the glycan binding profile of MN3. Glycan array analysis was performed on CFG microarray v5.3 with 50 µg/ml MN3. Results of a single glycan array experiment. Spots with highest and lowest fluorescence intensity (of the eight spots for each glycan) were removed. Error bars represent standard deviations. (G) Glycans found to be bound by MN3 (representative glycans out of the 42 glycans found be bound by MN3). Results of Glycopattern tool using two glycan array datasets of the same protein preparation at 5 and 50 µg/ml. (i-iii) Glycans with LacNAc unit with galactose in terminal position(s); (iv-vii) Glycans with fucose residues; (iv) Glycan with core fucose; (v) Glycan with type-2 H antigen; (v) Glycan with Lewis X antigen; (vi) Glycan with Lewis Y antigen; (viii) Bi-antennary glycan with one antenna having LacNAc and terminal Galactose and other antenna with terminal sialic acid; (ix) Glycan with terminal disialic acid sequences with α2-8 linkages. (H) Glycans found not to bind to MN3. Results of Glycopattern tool using two glycan array datasets of the same protein preparation at 5 and 50 µg/ml. (i-iii) Glycans with type-1 glycan unit; (iv-vi) Glycans with terminal acidic residues or GlcNAc and no galactose; (vii) Glycan with only one α2-8 linkage in the bi-antennary structure; (viii) Glycan with terminal galactose replaced by GalNAc; (ix) Glycan with terminal GlcNac. Monosaccharide symbols follow the SNFG (Symbol Nomenclature for Glycans) system (Varki, A., et al. 2015). (I) Glycan motifs identified by Motif Finder using automated model building function and default motif list and model options. Motif Finder analysis was performed using two glycan array datasets of the same protein preparation at 5 and 50 µg/ml. (J) Concentration dependent binding of MN3 to glycan motifs identified by Motif Finder. Results of Motif Finder analysis using two glycan array datasets of the same protein preparation at 5 and 50 µg/ml. (K) Bar plot showing the glycan binding profile of MU1. Glycan array analysis was performed on CFG microarray v5.3 with 50 µg/ml MU1. Results of a single glycan array experiment. Spots with highest and lowest fluorescence intensity (of the eight spots for each glycan) were removed. Error bars represent standard deviations. (L) Glycans found to be bound by MU1 (representative glycans out of the 64 glycans found to be bound by MU1). Results of Glycopattern tool using two glycan array datasets of the same protein preparation at 5 and 50 µg/ml. (i-ii) Glycans with terminal galactose residue(s); top two binders of MU1; they are also core fucosylated; (iii-iv) Glycans with fucose residue(s); (iii) Type-2 H antigen, (iv) Lewis-X antigen, (v) Lewis-Y antigen; (vi) Glycan with terminal disialic acid sequences with α2-8 linkages; (vii) Glycan with LacDiNAc units. (M) Glycans found not to bind to MU1. Results of Glycopattern tool using two glycan array datasets of the same protein preparation at 5 and 50 µg/ml. (i) Glycans with terminal GlcNAc residue; (ii) Glycans with type-1 glycan units. (iii) Glycan with terminal type-2 A antigen. (iv-v) Fucose containing glycans with short LacNAc

chains. Monosaccharide symbols follow the SNFG (Symbol Nomenclature for Glycans) system (Varki, A., et al. 2015). (N) Glycan motifs identified by Motif Finder using automated model building function and default motif list and model options. Motif Finder analysis was performed using two glycan array datasets of the same protein preparation at 5 and 50 µg/ml. (O) Concentration dependent binding of MU1 to glycan motifs identified by Motif Finder. Results of Motif Finder analysis using two glycan array datasets of the same protein preparation at 5 and 50 µg/ml. (P) Bar plot showing the glycan binding profile of MU3. Glycan array analysis was performed on CFG microarray v5.3 with 50 µg/ml MU3. Results of a single glycan array experiment. Spots with highest and lowest fluorescence intensity (of the eight spots for each glycan) were removed. Error bars represent standard deviations. (Q) Glycans found to be bound by MU3 (representative glycans out of the 11 glycans found tbe bound by MU3). Results of Glycopattern tool using two glycan array datasets of the same protein preparation at 5 and 50 µg/ml. (i-iv) All the glycans have terminal sialic acid residue(s) linked via α2-3 linkage; (R) Glycans found not to bind to MU3. Results of Glycopattern tool using two glycan array datasets of the same protein preparation at 5 and 50 µg/ml. (v-x) Glycans with terminal sialic acid residues linked via α2-6 linkage; (v-vi) Biantennary glycans with two LacNAc units on each arm; (vii) Glycans without more than one antenna. (viii-x) Glycans without sialic acid in α2-6 linkage in one of the two arms. Monosaccharide symbols follow the SNFG (Symbol Nomenclature for Glycans) system (Varki, A., et al. 2015). (S) Glycan motifs identified by Motif Finder using automated model building function and default motif list and model options. Motif Finder analysis was performed using two glycan array datasets of the same protein preparation at 5 and 50 µg/ml. (T) Concentration dependent binding of MU3 to glycan motifs identified by Motif Finder. Results of Motif Finder analysis using two glycan array datasets of the same protein preparation at 5 and 50 µg/ml.

Figure S5

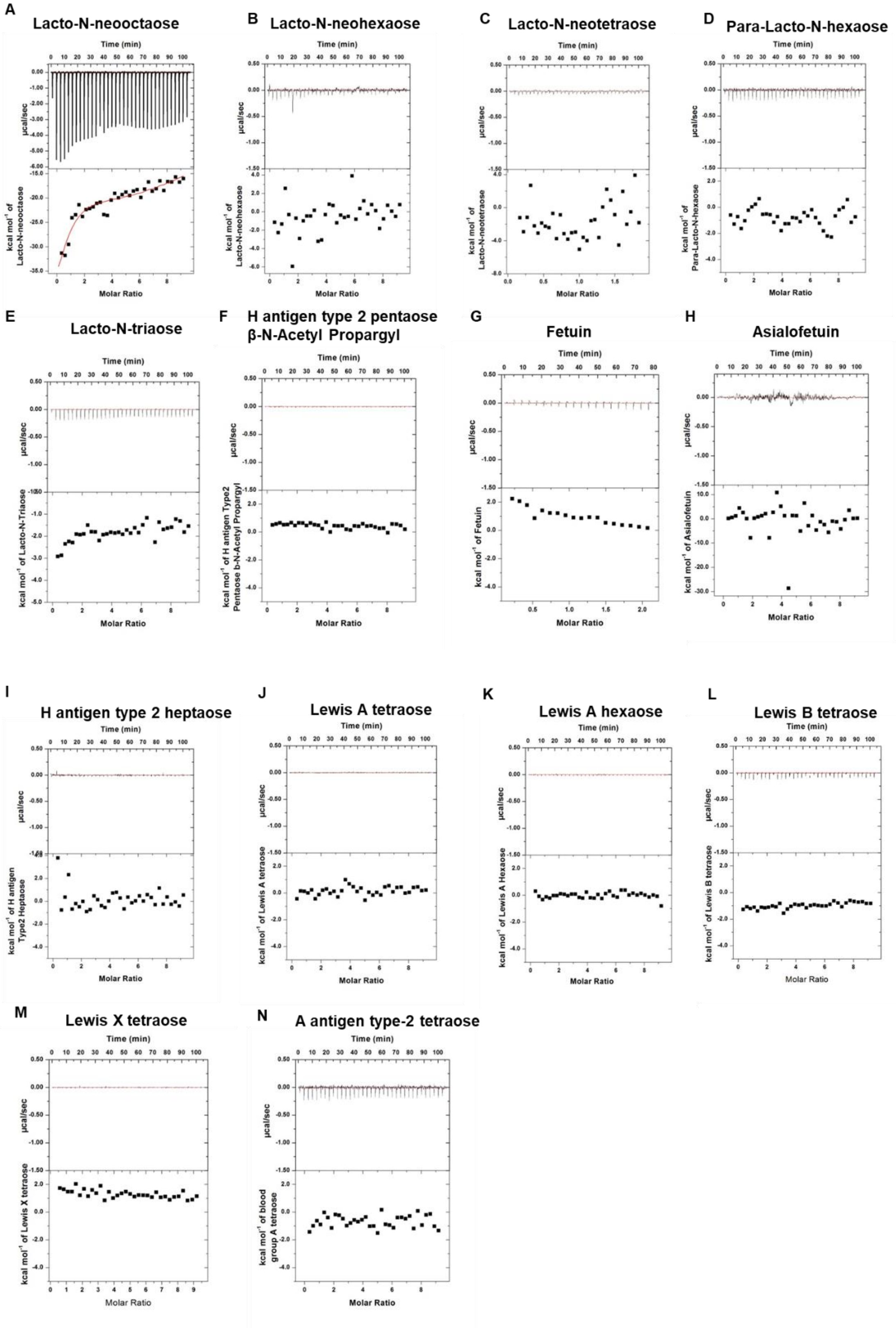

A
Lacto-N-neooctaose
B
Lacto-N-neohexaose
C
Lacto-N-neotetraose
D
Para-Lacto-N-hexaose
E
Lacto-N-triaose
F
H antigen type 2 pentaose β-N-Acetyl Propargyl
G
Fetuin
H
Asialofetuin
I
H antigen type 2 heptaose
J
Lewis A tetraose
K
Lewis A hexaose
L
Lewis B tetraose
M
Lewis X tetraose
N
A antigen type-2 tetraose
Time (min)
µcal/sec
Molar Ratio
kcal mol⁻¹ of Lacto-N-neooctaose
kcal mol⁻¹ of Lacto-N-neohexaose
kcal mol⁻¹ of Lacto-N-neotetraose
kcal mol⁻¹ of Para-Lacto-N-hexaose
kcal mol⁻¹ of Lacto-N-Triaose
kcal mol⁻¹ of H antigen Type2 Pentaose b-N-Acetyl Propargyl
kcal mol⁻¹ of Fetuin
kcal mol⁻¹ of Asialofetuin
kcal mol⁻¹ of H antigen Type2 Heptaose
kcal mol⁻¹ of Lewis A tetraose
kcal mol⁻¹ of Lewis A Hexaose
kcal mol⁻¹ of Lewis B tetraose
kcal mol⁻¹ of Lewis X tetraose
kcal mol⁻¹ of blood group A tetraose

O

**Ligand**	**Structure of Ligand**	**Protein concentration (mM)**	**Ligand concentration (mM)**	**Conclusion**
Gly023 (Lacto-N-neooctaose)	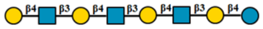	0.03	1.5	Inconclusive (High heats were observed in control reaction when Gly023 was injected into buffer in the cell)
Gly022 (Lacto-N-neohexaose)	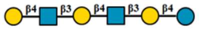	0.03	1.5	Binding not detected
Gly021 (Lacto-N-neotetraose)	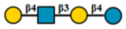	0.05	2.5	Binding not detected
Gly012 (Para-Lacto-N-hexaose)	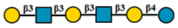	0.03	1.5	Binding not detected
Gly011 (Lacto-N-triaose) *	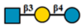	0.01	0.5	Binding not detected
Gly035-2 (A antigen type-2 tetraose)	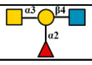	0.03	1.5	Binding not detected
Gly033-2NPR (H antigen pentaose type-2-β-N-Acetyl Propargyl) *	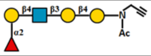	0.01	0.5	Binding not detected
Fetuin	Mainly triantennary oligosaccharides	0.015	0.5	Binding not detected
Asialofetuin	Mainly triantennary oligosaccharides without terminal sialic acid residues	0.015	0.5	Binding not detected
TE135 (H antigen type-2 heptaose azido ethyl) *	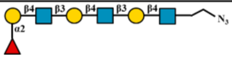	0.01	0.5	Binding not detected
Gly054 (Lewis A tetraose) *	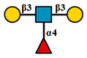	0.01	0.5	Binding not detected
Gly055 (Lacto-N-difucohexaose II) *	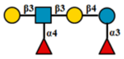	0.01	0.5	Binding not detected
Gly045 (Lewis B tetraose) *	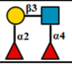	0.01	0.5	Binding not detected
Gly050 (Lewis X tetraose) *	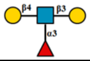	0.01	0.5	Binding not detected

Temperature of binding assays was 20 ^{o}C except for * where the temperature was 30 ^{o}C

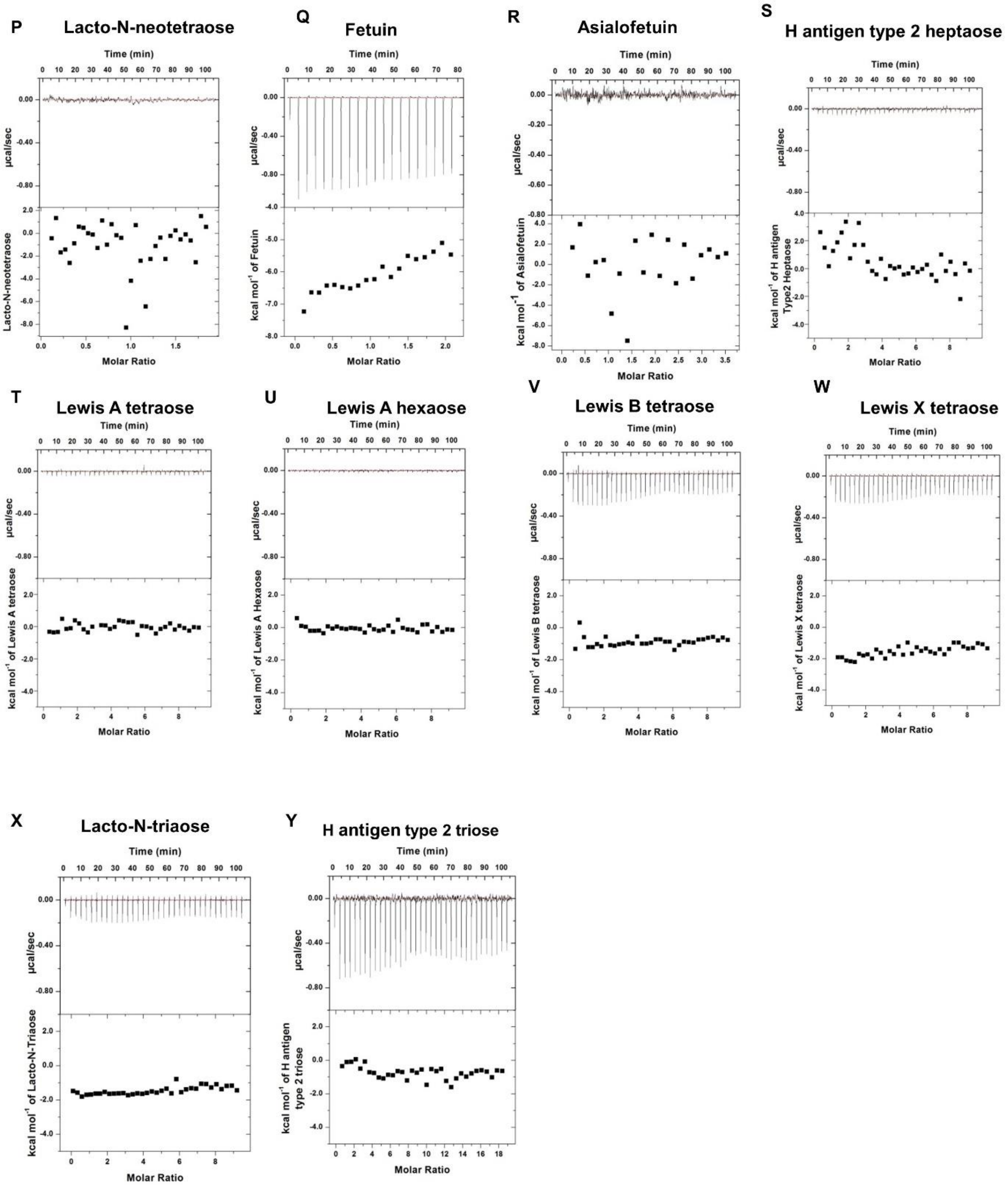
P
Lacto-N-neotetraose
Q
Fetuin
R
Asialofetuin
S
H antigen type 2 heptaose
T
Lewis A tetraose
U
Lewis A hexaose
V
Lewis B tetraose
W
Lewis X tetraose
X
Lacto-N-triaose
Y
H antigen type 2 triose
Time (min)
µcal/sec
Molar Ratio
kcal mol^{-1} of Lacto-N-neotetraose
kcal mol^{-1} of Fetuin
kcal mol^{-1} of Asialofetuin
kcal mol^{-1} of H antigen Type2 Heptaose
kcal mol^{-1} of Lewis A tetraose
kcal mol^{-1} of Lewis A Hexaose
kcal mol^{-1} of Lewis B tetraose
kcal mol^{-1} of Lewis X tetraose
kcal mol^{-1} of Lacto-N-Triaose
kcal mol^{-1} of H antigen type 2 triose

z

Ligand	Structure of Ligand	Protein concentration (mM)	Ligand concentration (mM)	Conclusion
Gly021 (Lacto-N-neotetraose)	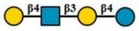	0.05	2.5	Binding not detected
Gly011 (Lacto-N-triaose) *	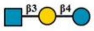	0.01	0.5	Binding not detected
Gly033-2NPR (H antigen pentaose type-2-β-N-Acetyl Propargyl)	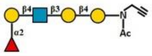	0.05	2.5	**Binding**
Gly031-2 (H Antigen type-2 triaose)	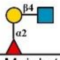	0.03	3	Binding not detected
Fetuin	Mainly triantennary oligosaccharides	0.05	0.5	Binding not detected
Asialofetuin	Mainly triantennary oligosaccharides without terminal sialic acid residues	0.015	0.5	Binding not detected
TE135 (H antigen type-2 heptaose azido ethyl) *	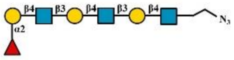	0.01	0.5	Binding not detected
Gly054 (Lewis A tetraose) *	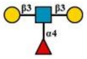	0.01	0.5	Binding not detected
Gly055 (Lacto-N-difucohexaose II) *	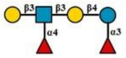	0.01	0.5	Binding not detected
Gly045 (Lewis B tetraose) *	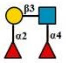	0.01	0.5	Binding not detected
Gly050 (Lewis X tetraose) *	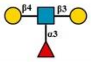	0.01	0.5	Binding not detected

Temperature of binding assays was 20 °C except for * where the temperature was 30 °C

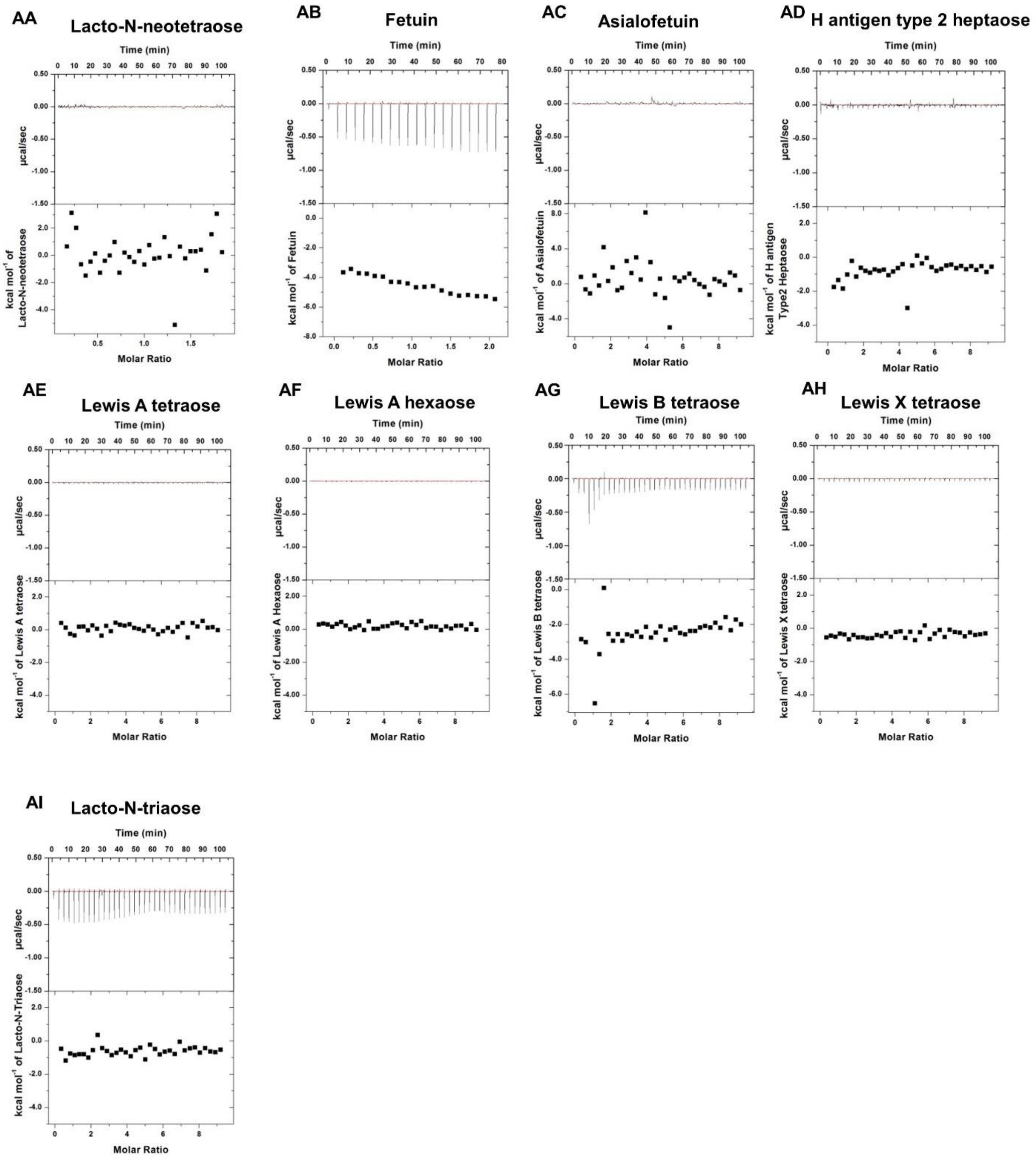

AA
Lacto-N-neotetraose
Time (min)
µcal/sec
kcal mol⁻¹ of Lacto-N-neotetraose
Molar Ratio
AB
Fetuin
Time (min)
µcal/sec
kcal mol⁻¹ of Fetuin
Molar Ratio
AC
Asialofetuin
Time (min)
µcal/sec
kcal mol⁻¹ of Asialofetuin
Molar Ratio
AD
H antigen type 2 heptaose
Time (min)
µcal/sec
kcal mol⁻¹ of H antigen Type2 Heptaose
Molar Ratio
AE
Lewis A tetraose
Time (min)
µcal/sec
kcal mol⁻¹ of Lewis A tetraose
Molar Ratio
AF
Lewis A hexaose
Time (min)
µcal/sec
kcal mol⁻¹ of Lewis A Hexaose
Molar Ratio
AG
Lewis B tetraose
Time (min)
µcal/sec
kcal mol⁻¹ of Lewis B tetraose
Molar Ratio
AH
Lewis X tetraose
Time (min)
µcal/sec
kcal mol⁻¹ of Lewis X tetraose
Molar Ratio
AI
Lacto-N-triaose
Time (min)
µcal/sec
kcal mol⁻¹ of Lacto-N-Triaose
Molar Ratio

AJ

Ligand	Structure of Ligand	Protein concentration (mM)	Ligand concentration (mM)	Conclusion
Gly021 (Lacto-N-neotetraose)	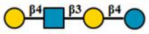	0.05	2.5	Binding not detected
Gly011 (Lacto-N-triaose) *	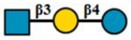	0.01	0.5	Binding not detected
Gly033-2NPR (H antigen pentaose type-2-β-N-Acetyl Propargyl)	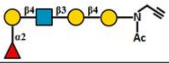	0.05	2.5	**Binding**
Fetuin	Mainly triantennary oligosaccharides	0.05	0.5	Binding not detected
Asialofetuin	Mainly triantennary oligosaccharides without terminal sialic acid residues	0.015	0.5	Binding not detected
TE135 (H antigen type-2 heptaose azido ethyl) *	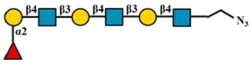	0.01	0.5	Binding not detected
Gly054 (Lewis A tetraose) *	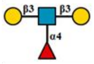	0.01	0.5	Binding not detected
Gly055 (Lacto-N-difucohexaose II) *	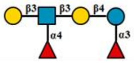	0.01	0.5	Binding not detected
Gly045 (Lewis B tetraose) *	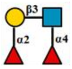	0.01	0.5	Binding not detected
Gly050 (Lewis X tetraose) *	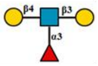	0.01	0.5	Binding not detected

Temperature of binding assays was 20 °C except for * where the temperature was 30 °C

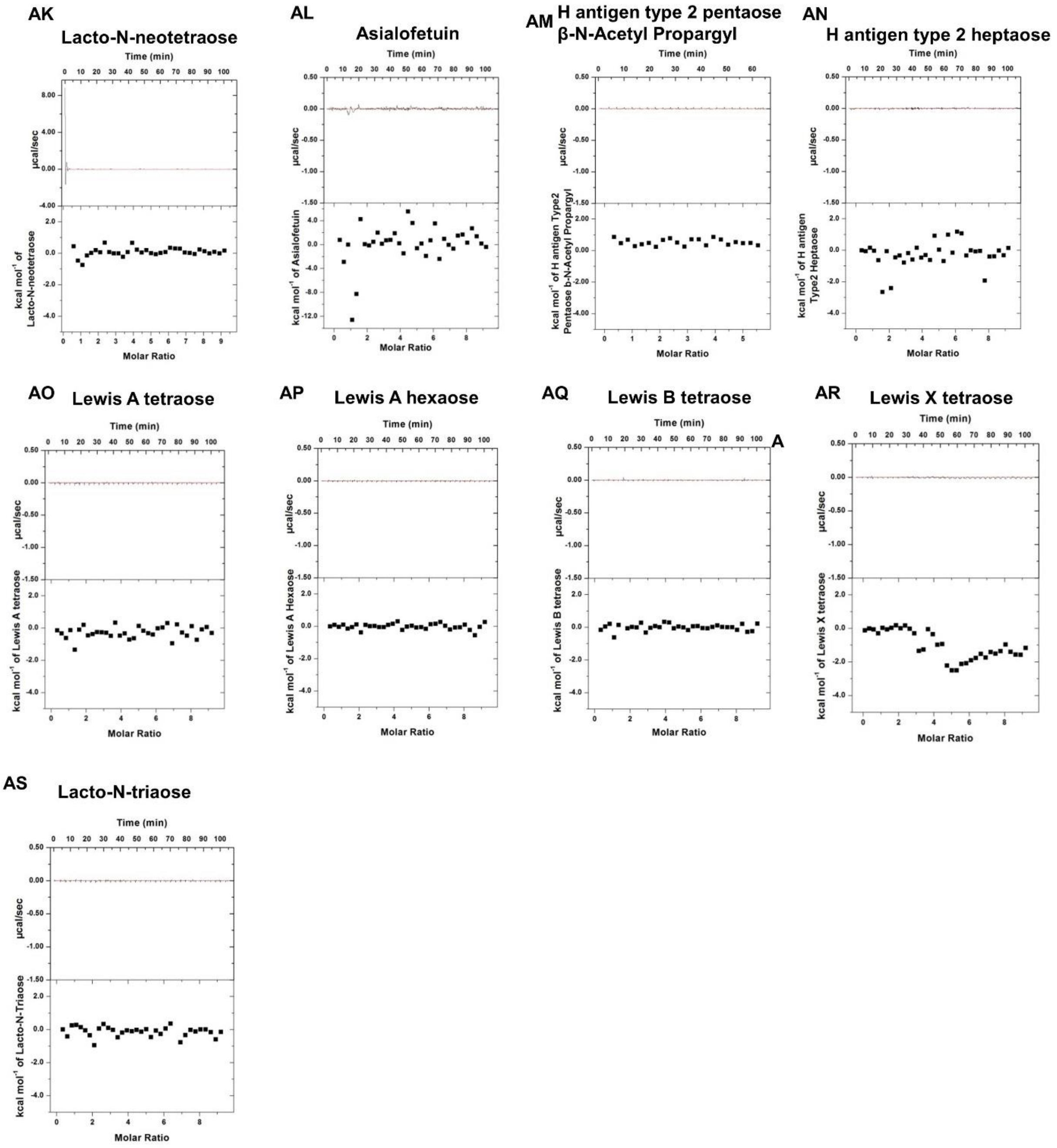
AK
Lacto-N-neotetraose
AL
Asialofetuin
AM
H antigen type 2 pentaose β-N-Acetyl Propargyl
AN
H antigen type 2 heptaose
AO
Lewis A tetraose
AP
Lewis A hexaose
AQ
Lewis B tetraose
AR
Lewis X tetraose
AS
Lacto-N-triaose
Time (min)
µcal/sec
Molar Ratio
kcal mol-1 of Lacto-N-neotetraose
kcal mol-1 of Asialofetuin
kcal mol-1 of H antigen Type2 Pentaose b-N-Acetyl Propargyl
kcal mol-1 of H antigen Type2 Heptaose
kcal mol-1 of Lewis A tetraose
kcal mol-1 of Lewis A Hexaose
kcal mol-1 of Lewis B tetraose
kcal mol-1 of Lewis X tetraose
kcal mol-1 of Lacto-N-Triaose

AT

Ligand	Structure of Ligand	Protein concentration (mM)	Ligand concentration (mM)	Conclusion
Gly021 (Lacto-N-neotetraose)	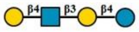	0.05	2.5	Binding not detected
Gly011 (Lacto-N-triaose) *	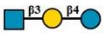	0.051	0.5	Binding not detected
Gly033-2NPR (H antigen pentaose type-2-β-N-Acetyl Propargyl) *	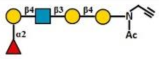	0.01	0.5	Binding not detected
Fetuin	Mainly triantennary oligosaccharides	0.05	0.5	**Binding**
Asialofetuin	Mainly triantennary oligosaccharides without terminal sialic acid residues	0.01	0.5	Binding not detected
TE135 (H antigen type-2 heptaose azido ethyl) *	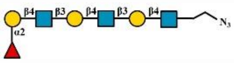	0.01	0.5	Binding not detected
Gly054 (Lewis A tetraose) *	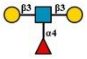	0.01	0.5	Binding not detected
Gly055 (Lacto-N-difucohexaose II) *	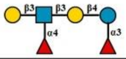	0.01	0.5	Binding not detected
Gly045 (Lewis B tetraose) *	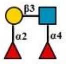	0.01	0.5	Binding not detected
Gly050 (Lewis X tetraose) *	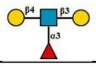	0.01	0.5	Binding not detected

Temperature of binding assays was 20 °C except for * where the temperature was 30 °C

Isothermal calorimetry of MG1 (A-O), MN3 (P-Z), MU1 (AA-AJ), MU3 (AK-AT). (A-N) Data for titrations of MG1 (loaded in the cell) with various glycans (loaded in the syringe) tabulated (O) as per the conditions mentioned. (P-Y) Data for titrations of MN3 (loaded in the cell) with various glycans (loaded in the syringe) tabulated (Z) as per the conditions mentioned. (AA-AI) Data for titrations of MU1 (loaded in the cell) with various glycans (loaded in the syringe) tabulated (AJ) as per the conditions mentioned. (AK-AS) Data for titrations of MU3 (loaded in the cell) with various glycans (loaded in the syringe) tabulated (AT) as per the conditions mentioned. All data shown are results of single ITC runs. Two control titrations were performed for each titration – glycan injected into buffer (in the cell), and buffer injected into protein (in the cell). The first injection was removed for all the plots. The control titration in which buffer was injected into protein solution was used as the control, and subtracted from the data prior to plotting, except for the titrations of MG1 and MU3 with Lacto-N-triaose, where the control titration in which sugar was injected into buffer solution was used to subtract the background. For the titration of MN3 with Lewis A tetraose, the data point corresponding to injection 13 was removed, and for the titration of MU3 with Lewis B tetraose, the data point corresponding to injection 7 was removed.

Figure S6

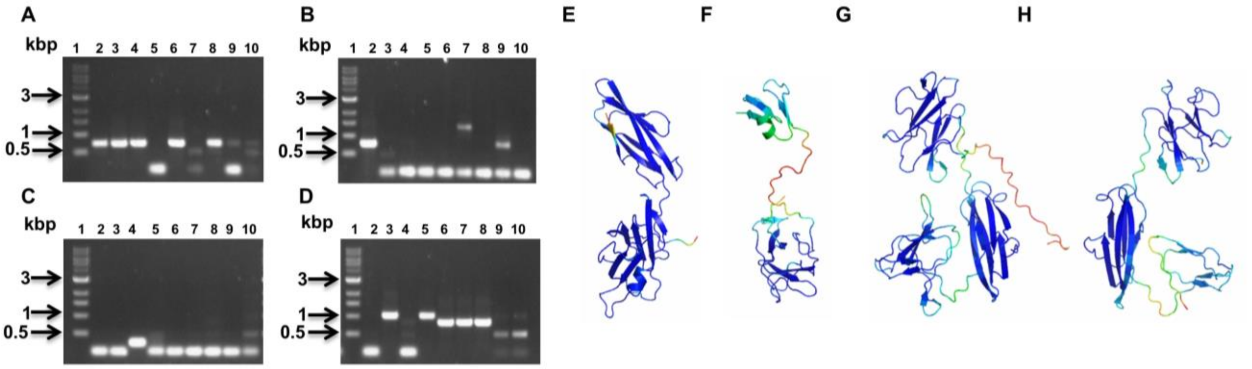

Identification of amylose and amylopectin binding protein domains. (A-D) Agarose gel electrophoresis of PCR amplicons indicating the presence or absence of any recombinant insert following biopanning of the metagenomic phage display library against amylose or amylopectin. (A) Biopanning against amylose and elution with D-glucose. Results of a single screening experiment. Lane 1: 1 kbp DNA marker. Lanes 2-9: PCR amplicons of clones Am-Glc1 to Am-Glc9. (B) Biopanning against amylose and elution with amylose. Results of a single screening experiment. Lane 1: 1 kbp DNA marker. Lanes 2-9: PCR amplicons of clones Am-Am1 to Am-Am9. (C) Biopanning against amylopectin and elution with D-glucose. Results of a single screening experiment. Lane 1: 1 kbp DNA marker. Lanes 2-9: PCR amplicons of clones Ap-Glc1 to Ap-Glc9. (D) Biopanning against amylopectin and elution with amylopectin. Results of a single screening experiment. Lane 1: 1 kbp DNA marker. Lanes 2-9: PCR amplicons of clones Ap-Ap1 to Ap-Ap9. (E-H) AlphaFold2 predicted structural models of Am-Glc2 (E), Am-Am1 (F), Ap-Ap2 (G), and Ap-Ap5 (H).

Figure S7

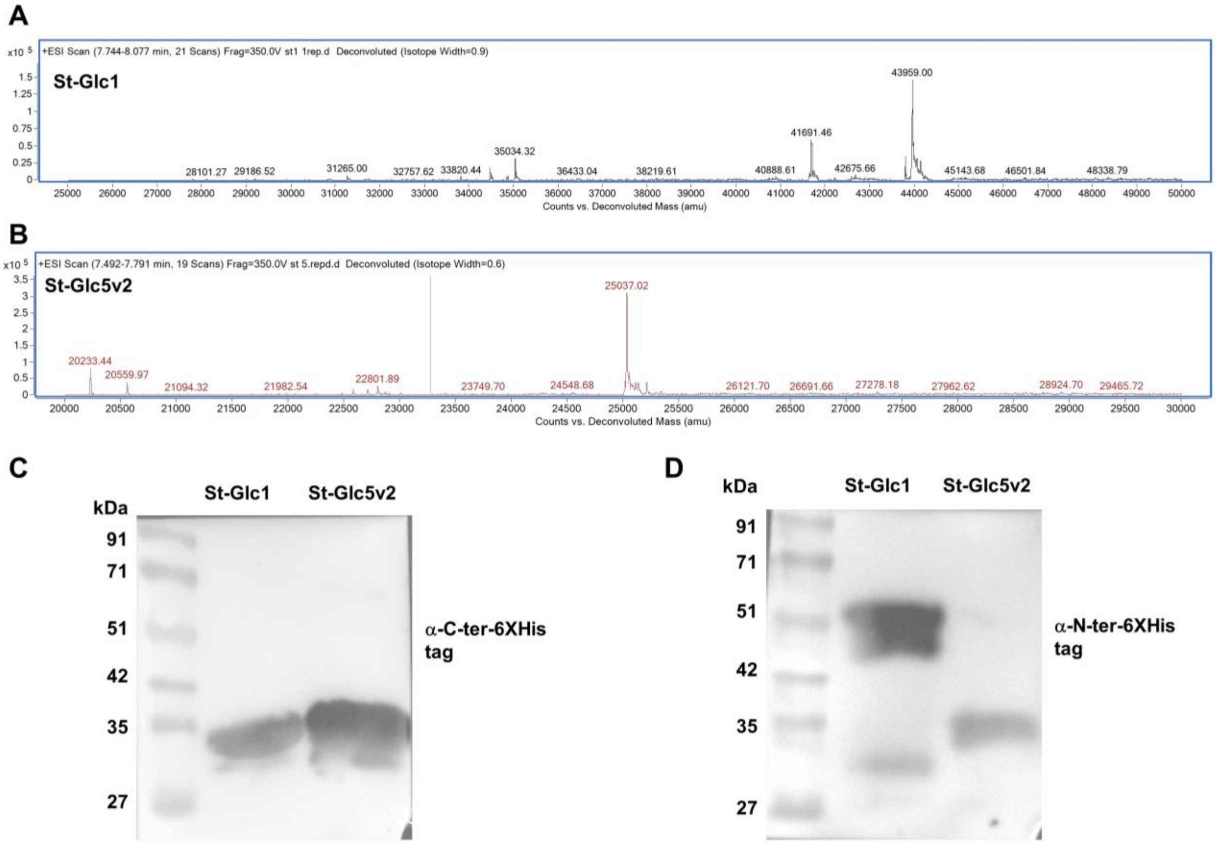

Recombinant St-Glc1 and St-Glc5v2 proteins. (A) Intact mass analysis of St-Glc1 by ESI-mass spectrometry. (B) Intact mass analysis of St-Glc5v2 by ESI-mass spectrometry. (C) Western analysis of St-Glc1 and St-Glc5v2 by anti-C-ter-6XHis tag antibody. (D) Western analysis of St-Glc1 and St-Glc5v2 by anti-N-ter-6XHis tag antibody.

Figure S8

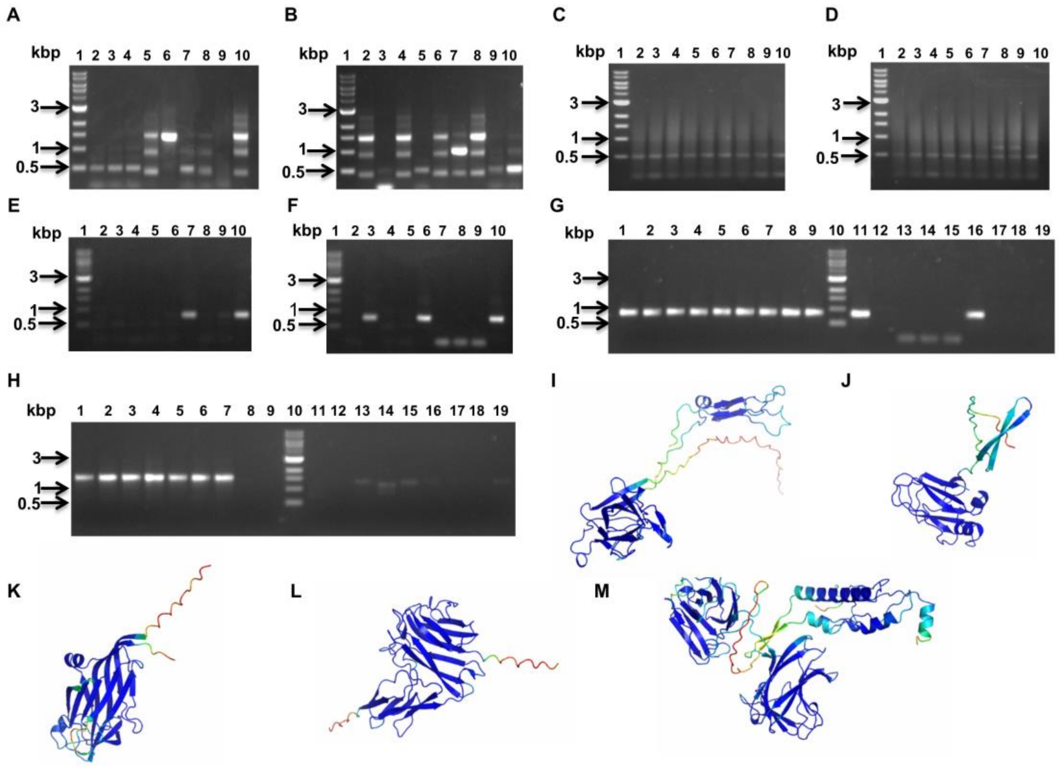

Identification of various carbohydrate binding protein domains. (A-J) Agarose gel electrophoresis of PCR amplicons indicating the presence or absence of any recombinant insert following biopanning of the metagenomic phage display library against various carbohydrates. (A) Biopanning against β-D-glucan and elution with D-glucose. Results of a single screening experiment. Lane 1: 1 kbp DNA marker. Lanes 2-9: PCR amplicons of clones BDG-Glc1 to BDG-Glc9. (B) Biopanning against β-D-glucan and elution with β-D-glucan. Results of a single screening experiment. Lane 1: 1 kbp DNA marker. Lanes 2-9: PCR amplicons of clones BDG-BDG1 to BDG-BDG9. (C) Biopanning against dextran and elution with D-glucose. Results of a single screening experiment. Lane 1: 1 kbp DNA marker. Lanes 2-9: PCR amplicons of clones Dex-Glc1 to Dex-Glc9. (D) Biopanning against dextran and elution with dextran. Results of a single screening experiment. Lane 1: 1 kbp DNA marker. Lanes 2-9: PCR amplicons of clones Dex-Dex1 to Dex-Dex9. (E) Biopanning against laminarin and elution with D-glucose. Results of a single screening experiment. Lane 1: 1 kbp DNA marker. Lanes 2-9: PCR amplicons of clones Lam-Glc1 to Lam-Glc9. (F) Biopanning against laminarin and elution with laminarin. Results of a single screening experiment. Lane 1: 1 kbp DNA marker. Lanes 2-9: PCR amplicons of clones Lam-Lam1 to Lam-Lam9. (G) Biopanning against pectin and elution with D-galacturonic acid or pectin. Results of a single screening experiment. Lanes 1-9: PCR amplicons of clones Pec-GalA1 to Pec-GalA9. Lane 10: 1 kbp DNA marker. Lanes 11-19: PCR amplicons of clones Pec-Pec1 to Pec-Pec9. (H) Biopanning against xylan and elution with D-xylose or xylan. Results of a single screening experiment. Lanes 1-9: PCR amplicons of clones Xln-Xyl1 to Xln-Xyl9. Lane 10: 1 kbp DNA marker. Lanes11-19: PCR amplicons of clones Xln-Xln1 to Xln-Xln9. (I-M) AlphaFold2 predicted structural models of BDG-Glc5 (I), BDG-BDG6 (J), Dex-Dex7 (K), Pec-GalA9 (L), and Xln-Xyl8 (M).

Figure S9

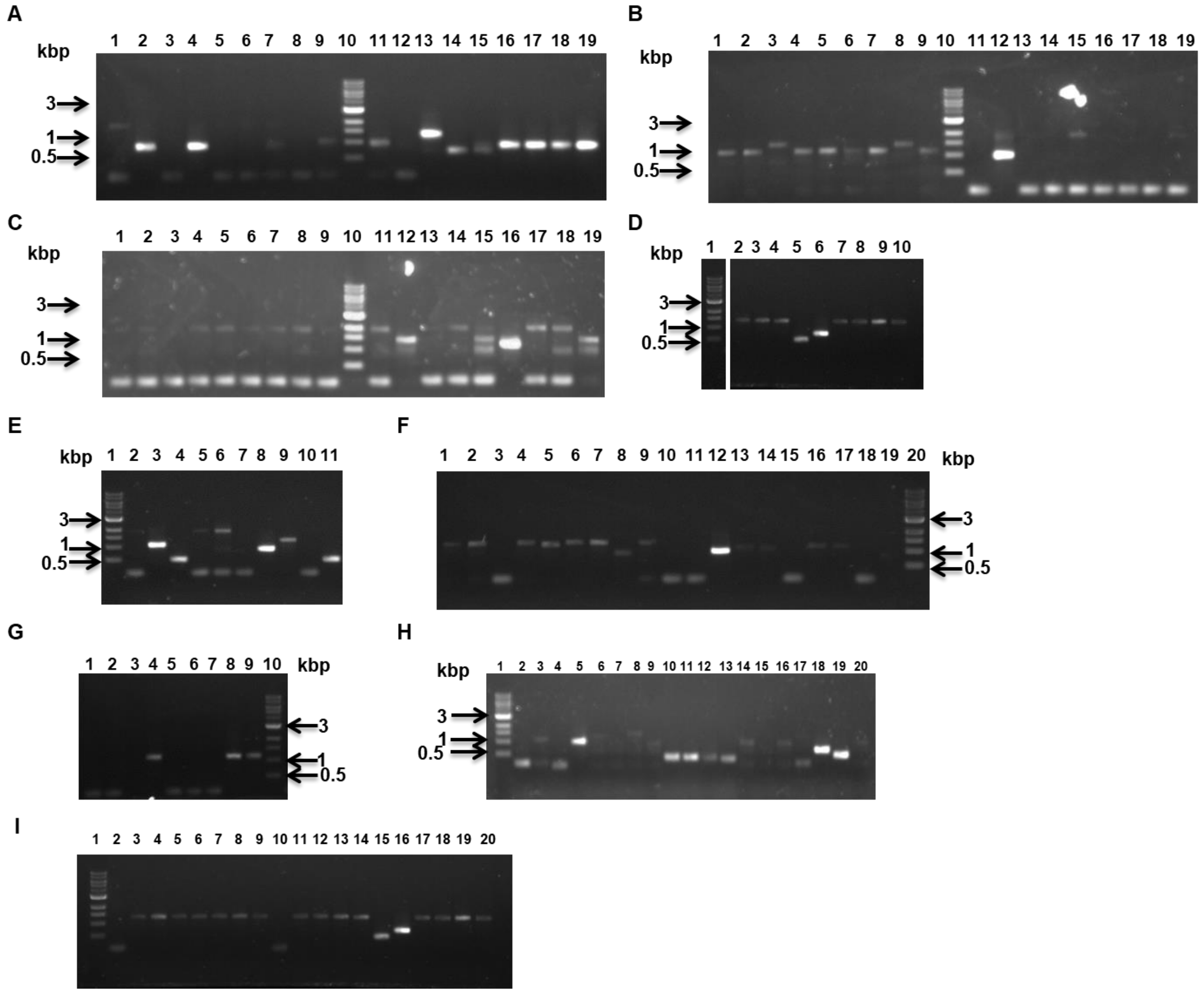

Identification of phage clones following screening against various carbohydrates. (A-J) Agarose gel electrophoresis of PCR amplicons indicating the presence or absence of any recombinant insert following biopanning of the metagenomic phage display library against various carbohydrates. (A) Biopanning against *Staphylococcus aureus* peptidoglycan and elution with GlcNAc or *Staphylococcus aureus* peptidoglycan. Results of a single screening experiment. Lanes 1-9: PCR amplicons of clones StapPG-GlcNAc1 to StapPG-GlcNAc9. Lane 10: 1 kbp DNA marker. Lanes 11-19: PCR amplicons of clones StapPG-StapPG1 to StapPG-StapPG9. (B) Biopanning against *Methanobacterium sp.* peptidoglycan and elution with GlcNAc or *Methanobacterium sp.* peptidoglycan. Results of a single screening experiment. Lanes 1-9: PCR amplicons of clones MethPG-GlcNAc1 to MethPG-GlcNAc9. Lane 10: 1 kbp DNA marker. Lanes 11-19: PCR amplicons of clones MethPG-MethPG1 to MethPG-MethPG9. (C) Biopanning against inulin and elution with D-fructose or D-glucose. Results of a single screening experiment. Lanes 1-9: PCR amplicons of clones Iln-Fru1 to Iln-Fru9. Lane 10: 1 kbp DNA marker. Lanes 11-19: PCR amplicons of clones Iln-Glc1 to Iln-Glc9. (D) Biopanning against inulin and elution with inulin. Results of a single screening experiment. Lane 1: 1 kbp DNA marker. Lanes 2-9: PCR amplicons

of clones Iln-Iln1 to Iln-Iln9. (E) Biopanning against arabinogalactan and elution with D-galactose. Results of a single screening experiment. Lane 1: 1 kbp DNA marker. Lanes 2-10: PCR amplicons of clones AG-Gal1 to AG-Gal9. (F) Biopanning against arabinogalactan and elution with L-arabinose or arabinogalactan. Results of a single screening experiment. Lanes 1-9: PCR amplicons of clones AG-Ara1 to AG-Ara9. Lanes 10-19: PCR amplicons of clones AG-AG1 to AG-AG9. Lane 20: 1 kbp DNA marker. (G) Biopanning against PAA-b-D-Galactose and elution with D-galactose. Results of a single screening experiment. Lanes 1-9: PCR amplicons of clones PBGal-Gal1 to PBGal-Gal9. Lane 10: 1 kbp DNA marker. (H) Biopanning against PAA-b-D-GlcNAc and elution with D-Glucose. Results of a single screening experiment. Lane 1: 1 kbp DNA marker. Lanes 2-20: PCR amplicons of clones PBGlcNAc-Glc1 to PBGlcNAc-Glc9. (I) Uncropped image pertaining to S9D. Lane 1 is the molecular weight marker, and lanes 12 to 20 are lanes 2 to 10 in Figure S9D.

Figure S10

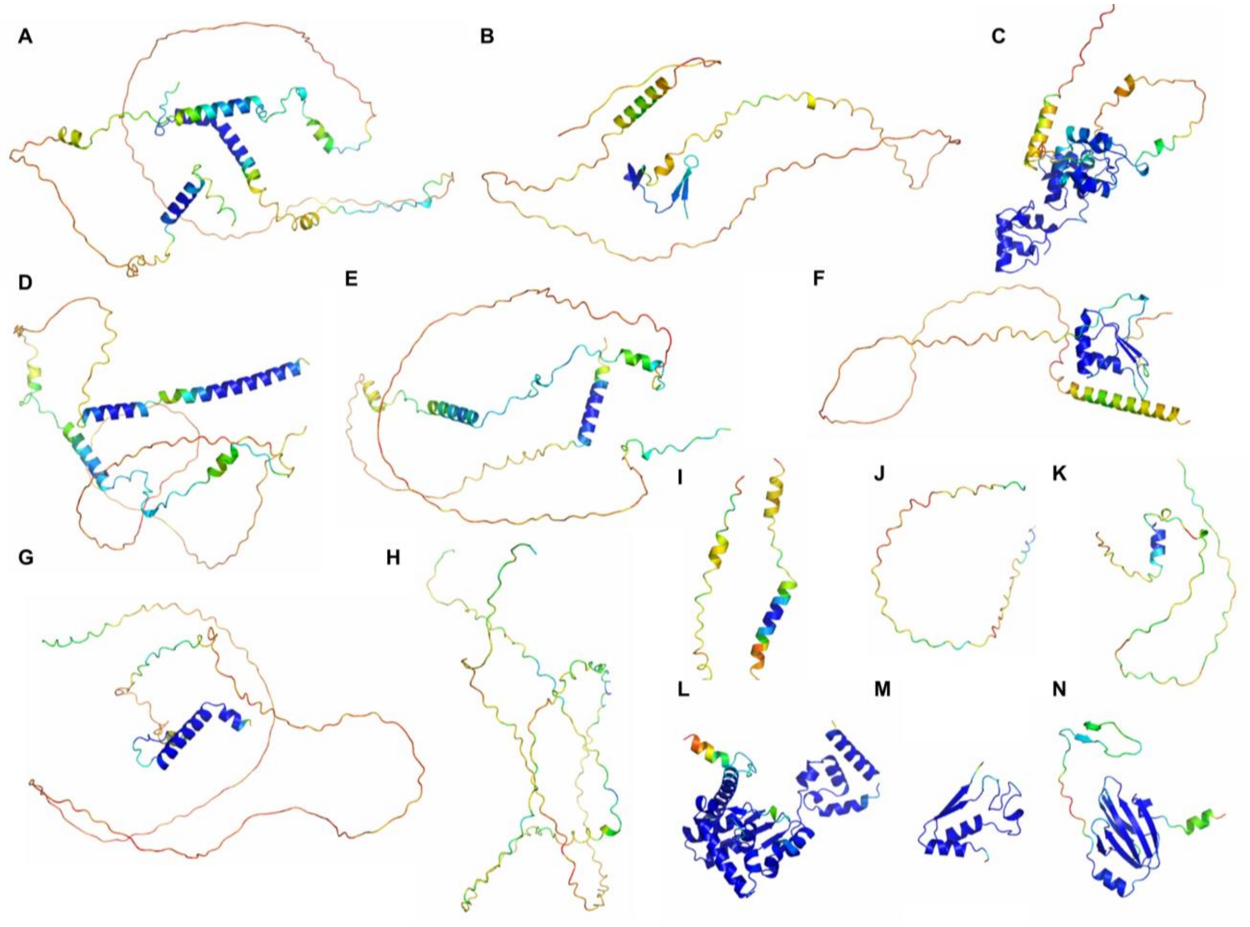

AlphaFold2 predicted structural models. (A) AG-Gal2. (B) Iln-Glc6. (C) PBGal-Gal8. (D) MethPG-MethPG8. (E) StapPG-GlcNAc6. (F) StapPG-StapPG6. (G) StapPG-StapPG7. (H) PBGlcNAc-GlcNAc4F1. (I) PBGlcNAc-GlcNAc8. (J) PBGlcNAc-GlcNAc9F1. (K) PBGlcNAc-GlcNAc17F1. (L) PBGlcNAc-GlcNAc4F2. (M) PBGlcNAc-GlcNAc9F2. (N) PBGlcNAc-GlcNAc17F2.

Figure S11

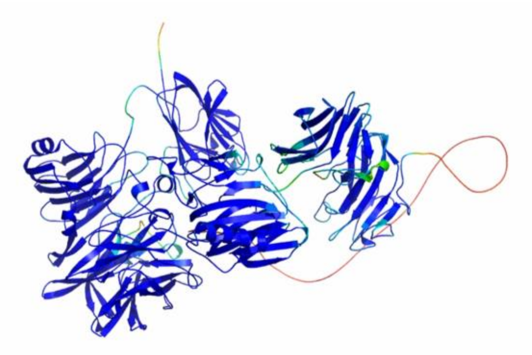

AlphaFold2 predicted structural model of *Paenibacillus macerans* (*Bacillus macerans*) protein (GenBank: AAG47946.1) with CBM38 and GH32 cycloinulooligosaccharide fructanotransferase domains.
