# Supplementary material for: New carbohydrate binding domains identified by phage display based functional metagenomic screens of human gut microbiota
Source: Commun Biol. 2023 Apr 5;6:371. doi: 10.1038/s42003-023-04718-0 (PMC10076258; doi:10.1038/s42003-023-04718-0)
Supplement: Supplementary file 3 — Description of Additional Supplementary Data [file 42003_2023_4718_MOESM3_ESM.docx]

**Description of Additional Supplementary Files**

**File name:** Supplementary Data 1

**Description:** Supplementary Tables S1 to S10

Table S1: List of ligands and elution reagents used in biopanning

Table S2: Titer of the eluate containing phages in various rounds of biopanning using 1x103: 1x1011 ratio of SrNaFLD-T7 phage: T7 10-3b (non-recombinant) phage

Table S3: List of the titers of the eluates obtained after screening against various glycans/glycoconjugates and eluting with different reagents from the 8th round of biopanning

Table S4: Sequences of metagenomic inserts in screened phages

Table S5: Search_Result_UniRef100_sensitivity_8.5.m8_processed

Table S6: Search_Result_dbCAN2_Sensitivity_8.5

Table S7: Search_Result_pfamA_Full_Sensitivity_8.5

Table S8: Search_Result_pfamB_sensitivity_8.5

Table S9: Search_Result_All_Against_All_Input_Sensitivity_8.5

Table S10: Glycan array data for MG1, MN3, MU1, and MU3

**File name:** Supplementary Data 2

**Description:** A zipped folder containing .pdb files and a Pymol session file of all the AlphaFold2 predicted structures of the proteins discussed in the paper

**File name:** Supplementary Data 3

**Description:** The source data behind the plots in the main figures of the paper
