# Supplementary material for: New carbohydrate binding domains identified by phage display based functional metagenomic screens of human gut microbiota
Source: Commun Biol. 2023 Apr 5;6:371. doi: 10.1038/s42003-023-04718-0 (PMC10076258; doi:10.1038/s42003-023-04718-0)
Supplement: Supplementary file 7 — Reporting Summary [file 42003_2023_4718_MOESM7_ESM.pdf]

nature portfolio

Corresponding author(s): T. N. C. Ramya

Last updated by author(s): 3/3/2023

Reporting Summary

Nature Portfolio wishes to improve the reproducibility of the work that we publish. This form provides structure for consistency and transparency in reporting. For further information on Nature Portfolio policies, see our Editorial Policies and the Editorial Policy Checklist.

Statistics

For all statistical analyses, confirm that the following items are present in the figure legend, table legend, main text, or Methods section.

n/a	Confirmed	
☐	☒	The exact sample size (*n*) for each experimental group/condition, given as a discrete number and unit of measurement
☐	☒	A statement on whether measurements were taken from distinct samples or whether the same sample was measured repeatedly
☒	☐	The statistical test(s) used AND whether they are one- or two-sided<br>*Only common tests should be described solely by name; describe more complex techniques in the Methods section.*
☒	☐	A description of all covariates tested
☒	☐	A description of any assumptions or corrections, such as tests of normality and adjustment for multiple comparisons
☐	☒	A full description of the statistical parameters including central tendency (e.g. means) or other basic estimates (e.g. regression coefficient) AND variation (e.g. standard deviation) or associated estimates of uncertainty (e.g. confidence intervals)
☒	☐	For null hypothesis testing, the test statistic (e.g. *F*, *t*, *r*) with confidence intervals, effect sizes, degrees of freedom and *P* value noted<br>*Give P values as exact values whenever suitable.*
☒	☐	For Bayesian analysis, information on the choice of priors and Markov chain Monte Carlo settings
☒	☐	For hierarchical and complex designs, identification of the appropriate level for tests and full reporting of outcomes
☒	☐	Estimates of effect sizes (e.g. Cohen's *d*, Pearson's *r*), indicating how they were calculated

Our web collection on statistics for biologists contains articles on many of the points above.

Software and code

Policy information about availability of computer code

Data collection	No code was developed for this analysis
Data analysis	No code was developed for this analysis

For manuscripts utilizing custom algorithms or software that are central to the research but not yet described in published literature, software must be made available to editors and reviewers. We strongly encourage code deposition in a community repository (e.g. GitHub). See the Nature Portfolio guidelines for submitting code & software for further information.

Data

Policy information about availability of data

All manuscripts must include a data availability statement. This statement should provide the following information, where applicable:

- Accession codes, unique identifiers, or web links for publicly available datasets
- A description of any restrictions on data availability
- For clinical datasets or third party data, please ensure that the statement adheres to our policy

A data availability statement is provided. Nucleotide sequences were submitted to NCBI. Accession numbers of all the sequences mentioned in the manuscript are listed in the supplementary table (Table S4) (MN313892, MN313893, MN313894, MN313895, ON711254, ON711255, ON711256, ON711257, ON711258, ON711259, ON711260, ON711261, ON711262, ON711263, ON711264, ON711265, ON711266, ON711267, ON711268, ON711269, ON711270, ON711271, ON711272, ON711273, ON711274, ON711275, ON711276, ON711277, ON711278, and ON711279). The metagenomic phage display library and the plasmids

described in the study are available upon request and payment of shipping charges; however, individual phage clones described in the study are no longer viable, and the diversity of the phage display library is likely to have decreased with the passage of time during storage. This has been mentioned in the data availability statement.

Human research participants

Policy information about studies involving human research participants and Sex and Gender in Research.

Reporting on sex and gender	There is no relevance of sex and gender in this study.
Population characteristics	Self-reported healthy individuals in the age group of 18-58 years of either sex and gender.
Recruitment	Subjects were self-reported healthy individuals in the age group of 18-58 years.
Ethics oversight	This study was approved by Council of Scientific and Industrial Research (CSIR)-Institute of Microbial Technology Institutional Ethics Committee (Human) (Project number 11 IEC/1/9-2014) and Council of Scientific and Industrial Research (CSIR)- Institute of Microbial Technology Institutional Biosafety Committee (Project number IBSC/2012-2/21). Written informed consent was obtained to release the information obtained as a result of the subject's participation and to publish the study while keeping the identity confidential.

Note that full information on the approval of the study protocol must also be provided in the manuscript.

Field-specific reporting

Please select the one below that is the best fit for your research. If you are not sure, read the appropriate sections before making your selection.

☒ Life sciences ☐ Behavioural & social sciences ☐ Ecological, evolutionary & environmental sciences

For a reference copy of the document with all sections, see nature.com/documents/nr-reporting-summary-flat.pdf

Life sciences study design

All studies must disclose on these points even when the disclosure is negative.

Sample size	N/A
Data exclusions	N/A
Replication	Biochemical assays were replicated. Screening with Phage Display Library was performed only once because our goal was to find the carbohydrate binding sequences, and later confirm the binding by performing biochemical assays.
Randomization	N/A
Blinding	N/A

Reporting for specific materials, systems and methods

We require information from authors about some types of materials, experimental systems and methods used in many studies. Here, indicate whether each material, system or method listed is relevant to your study. If you are not sure if a list item applies to your research, read the appropriate section before selecting a response.

Materials & experimental systems

n/a	Involved in the study	
☒	☐	Antibodies
☒	☐	Eukaryotic cell lines
☒	☐	Palaeontology and archaeology
☒	☐	Animals and other organisms
☒	☐	Clinical data
☒	☐	Dual use research of concern

Methods

n/a	Involved in the study	
☒	☐	ChIP-seq
☒	☐	Flow cytometry
☒	☐	MRI-based neuroimaging
